# SIRT1/SIRT3 Modulates Redox Homeostasis during Ischemia/Reperfusion in the Aging Heart

**DOI:** 10.3390/antiox9090858

**Published:** 2020-09-13

**Authors:** Jingwen Zhang, Di Ren, Julia Fedorova, Zhibin He, Ji Li

**Affiliations:** 1 College of Life Sciences, Shandong Normal University, Jinan 250014, China; jzhang14@usf.edu; 2 Department of Surgery, Morsani College of Medicine, University of South Florida, Tampa, FL 33612, USA; diren@usf.edu (D.R.); jfedorova@usf.edu (J.F.); zhibin@usf.edu (Z.H.)

**Keywords:** ischemia and reperfusion, SIRT1, SIRT3, ROS, aging heart

## Abstract

Ischemia/reperfusion (I/R) injury is the central cause of global death in cardiovascular diseases, which is characterized by disorders such as angina, stroke, and peripheral vascular disease, finally causing severe debilitating diseases and death. The increased rates of morbidity and mortality caused by I/R are parallel with aging. Aging-associated cardiac physiological structural and functional deterioration were found to contribute to abnormal reactive oxygen species (ROS) production during I/R stress. Disturbed redox homeostasis could further trigger the related signaling pathways that lead to cardiac irreversible damages with mitochondria dysfunction and cell death. It is notable that sirtuin proteins are impaired in aged hearts and are critical to maintaining redox homeostasis via regulating substrate metabolism and inflammation and thus preserving cardiac function under stress. This review discussed the cellular and functional alterations upon I/R especially in aging hearts. We propose that mitochondria are the primary source of reactive oxygen species (ROS) that contribute to I/R injury in aged hearts. Then, we highlight the cardiomyocyte protection of the age-related proteins Sirtuin1 (SIRT1) and Sirtuin1 (SIRT3) in response to I/R injury, and we discuss their modulation of cardiac metabolism and the inflammatory reaction that is involved in ROS formation.

## 1. Introduction

Ischemic heart disease, symbolized by the constriction in the coronary blood vessel, is one of the most significant cardiac problems with a higher death rate among the elderly population [1]. Compared to adult hearts, aged hearts are more vulnerable to ischemic insults and sustain greater injury during ischemia/reperfusion (I/R) [2]. In clinical, aging augmented in vivo reactive oxygen species (ROS) levels in acute myocardial infarction (AMI) patients [2]. The findings of proteomics analyses showed that the downregulation of elevated mitochondrial ROS levels protects old mice against age-related decline, supporting the theory that decreased ROS levels could be a beneficial factor on the extension of life span in the elder population [3,4,5]. Uncontrolled cardiac ROS generation caused by pathological alterations involved in myocardial I/R injury can promote oxidative damage to cellular proteins and other biomolecules, as well as mitochondrial dysfunction and cell death [6]. These discoveries advance the understanding of the mechanisms of abnormal redox homeostasis in order to develop potentially effective approaches to protect hearts from I/R injury, especially in the older population.

Sirtuins are a family of highly conserved proteins with homology to the yeast silent information regulator 2 (SIR2). Growing studies have implicated that sirtuins play vital roles in delaying cellular senescence and extending mammal lifespan [7]. Since they were identified in mammals, sirtuins have been implicated in many essential cellular processes and functions, including longevity [8], DNA damage repair [9], metabolism [10], and inflammation [11]. Sirtuin1 (SIRT1) has an evolutionary relationship to SIR2 and has been most intensively investigated in the cardiovascular system with effective deacetylase activity controlling cellular processes such as cell apoptosis, autophagy, and cell proliferation [12,13]. SIRT1 can diminish oxidative stress by activating cardioprotective molecules and inhibit apoptosis-related signaling pathways against cardiac I/R [14]. Sirtuin3 (SIRT3), as the central control of mitochondrial protein deacetylation, protects cardiomyocytes from aging and oxidative stress [15,16,17,18]. SIRT3 deficiency aggravates the cardiac susceptibility to I/R stress with severe mitochondria abnormalities and exacerbates a higher level of myocardial I/R injury with aging [19,20].

In this review, we will mainly focus on the functions of age-related sensors SIRT1 and SIRT3 via modulating redox homeostasis in response to myocardial I/R stress. This makes them potential targets for developing better cell-based therapeutic strategies for elderly patients against I/R-induced injury.

## 2. Role of Longevity Gene Sirtuins in Myocardial I/R

Sirtuins are mammalian homologs of yeast Sir2, a silent information regulator 2, which has the capacity to deacetylase numerous proteins in a nicotinamide adenine dinucleotide (NAD^+^)-dependent manner [10,21]. To date, seven members of the sirtuin family have been reported in mammals with various enzymatic activity and subcellular localization. All of them have the common catalytic domain that consists of 275 amino acids accounting for protein deacetylation [22,23]. In addition, ADP ribosylation is the main activity for Sirtuin4 (SIRT4) instead of deacetylase activity, which is also typical for Sirtuin6 (SIRT6) [24,25]. In addition, Sirtuin5 (SIRT5) has weak deacetylase activity but effective demalonylase and desuccinylase activity [26,27].

SIRT1, SIRT6, and Sirtuin7 (SIRT7) localize predominately in the nucleus, and more evidence regarding SIRT1 and SIRT6 but not SIRT7 are reported to imply important functional links to aging [28,29]. The elimination of SIRT1 expression causes only 50% of individuals to be born and only 20% to survive to maturity in mouse models. Such mice have developmental defects with phenotypic abnormalities in the eye and heart [30]. Furthermore, overexpressing SIRT1 in the mice hypothalamus can increase the lifespan by 16% in females and 9% in males [31]. Sirtuin2 (SIRT2) can be found mostly in the cytoplasm, and SIRT3, SIRT4, and SIRT5 are located in mitochondria [23]. SIRT3 is thought to be the central control of mitochondrial protein deacetylation due to the hyperacetylation of global mitochondrial proteins in the absence of SIRT3, but not SIRT4 or SIRT5 [32]. More interestingly, SIRT3 is the only sirtuin that has been reported to impact longevity in humans based on the fact that a certain polymorphism in the SIRT3 gene can be found more often in elder people [33,34]. SIRT3 has exhibited the capacity to reverse aging-associated degeneration via the control of mitochondrial homeostasis consistently [35]. Taken together, these results support that sirtuins play critical roles in maintaining mammalian longevity.

Sirtuins have beneficial roles in regulating cellular homeostasis with aging in a wide range of cardiovascular diseases including myocardial I/R [10]. For example, SIRT1 possesses many capabilities to protect hearts from myocardial I/R injury, including maintaining redox and metabolic homeostasis [36], repressing inflammatory reaction [37], inhibiting apoptosis [14], and promoting autophagy [38]. In our I/R model of cardiac specific SIRT1 knockout mice, the heart showed significantly enlarged infarction size, metabolic disorder, and excessive cardiac ROS levels [36]. We also found that the deletion of SIRT1 in cardiomyocytes caused the hyperacetylation of liver kinase B1 (LKB1) and impaired the phosphorylation of adenosine monophosphate protein kinase (AMPK) during ischemia [36]. These promote the significance of SIRT1 in the repression of energetic consuming processes via LKB1/AMPK during I/R stress. Moreover, SIRT1 deficiency leads to inflammatory-like phenotype alterations in the heart during I/R as well as upregulated inflammatory cytokines such Interleukin-1β (IL-1β), Tumor necrosis factoas-α (TNF-α) and Interleukin-6 (IL-6) during I/R injury [14,37], which is caused by the activation of the Nod-like receptor protein-3 (NLRP3) inflammasome. SIRT1 is also a potential target for cardiac apoptosis during I/R. It has been proven that the SIRT1-induced inhibition of p53 transcription is closely involved in the survival of cardiomyocytes, since p53-mediated apoptosis was activated as a result of the decreased SIRT1 during cardiac ischemia [14,39]. Furthermore, SIRT1-related autophagic regulation has been recognized to maintain cardiac function as the result of the deacetylation of autophagy-related protein (ATG) family members, such as ATG5, ATG7 and ATG8, and forkhead box class O protein (FoxO) [38].

The underlying role of SIRT3 in the alleviation of I/R injury in the heart is mainly related to the post-translational modification of mitochondrial bioenergetic processes [40]. Defected cardiac SIRT3 level causes an upregulation of ROS generation and the hyperacetylation of proteins associated with mitochondria oxidative phosphorylation (OXPHOS), fatty acid oxidation, and the tricarboxylic acid (TCA) cycle, as well as oxidative stress [20]. Recent research revealed the three ways that SIRT3 regulates cardiac autophagy during I/R stress, which are autophagosome formation-related AMPK/mTOR activation, the Foxo3a-mediated Pink1/Parkin pathway, and mitochondrial ROS homeostasis via superoxide dismutase 2 (SOD2) [41,42]. In this way, SIRT1 and SIRT3 have multiple beneficial effects on protecting hearts against I/R injury and suggest the significance of them as potential targets for cardioprotection, especially in the elder population. Considering the importance of ROS, which are primary toxic by-products of aerobic metabolism that lead to macromolecular damage in cardiovascular disease [6], this review focuses on summarizing the latest evidence regarding the role of SIRT1 and SIRT3 in redox homeostasis during cardiac I/R stress.

## 3. Reactive Oxygen Species in Age-Related Ischemic Heart Disease

ROS consist of unstable molecules containing oxygen with high instability and a short half-life [43]. They are physiologically related to regulate cellular homeostasis and mediate signal transduction for cardiac development, contractile function, and calcium handling [44]. Cardiac mitochondria are significantly abundant, and the high adenosine triphosphate (ATP) consumption of the beating heart depends on the oxidative energy generated by the mitochondria electron transport chain (ETC) [45]. Mitochondria ETC is the major endogenous source of the ROS (especially superoxide and hydroxyl radical) [46,47], especially complex I and III [48]. Complex I, as first enzyme of mitochondria ETC, catalytically transfers two electrons from the nicotinamide adenine dinucleotide hydride (NADH) matrix to coenzyme Q (CoQ) and leaks electrons to O_2_ [49]. The crystal structure of the hydrophilic domain in complex I [50] reveals that most of the cofactors in the enzyme are shielded from solvent. Therefore, it is most likely that O_2_ accesses complex I and produces superoxide through the two sites, site I_F_ (FMN site) and site I_Q_ (CoQ binding site), and then releases ROS to the matrix [51,52]. Complex III is another critical place for electron leakage through the Q-cycle resulting in superoxide production in the Qo site and releasing ROS into both the mitochondrial matrix and intermembrane space [53,54,55]. In this process, ubisemiquinone in the Qo site carries a single electron and directly transfers to O_2_ in complex III, generating superoxide via a nonenzymatic reaction [49,56].

Mitochondrial dysfunction, especially the impairment of ETC, is one of the important factors involved in cardiac dysfunctions induced by I/R injury. Upon myocardial ischemia, limited oxygen availability is intimately related to the mitochondrial OXPHOS arrest, leading to dramatically reduced ATP synthesis [57,58]. In order to counterbalance this situation, the troubled cardiomyocytes switch to anaerobic glycolysis to meet the metabolic demand accompanied with the aggregation of protons and lactate, eventually resulting in decreasing the intracellular pH [59] and promoting calcium overload in the cell [60,61]. ROS generation is associated with increased electron leakage as a result of impaired ETC, especially within three minutes of ischemia [62,63]. Ischemia directly increased the production of oxyradicals generated by complex I with compromised activity in the first 10–20 min [64]. During ischemia, the functional change of the Qo site in complex III can disturb the electron flux, leading to increased ROS production [65]. Reperfusion was characterized by a sudden restoration of oxygen delivery, and the ATP generation was rapidly switched from anaerobic glycolysis to aerobic mitochondrial OXPHOS with removing accumulated H+ in the extracellular space [66,67]. Although reperfusion intervention is the most effective strategy to salvage the ischemic heart [68,69,70], there are still complications such as the generation of ROS, cytokines, and chemokines exacerbating injury via the accumulation of cellular damage and mitochondrial abnormalities [71,72]. I/R causes substantial mitochondria swelling and cell death, altered cardiac metabolism such as glycolysis disturbance and TCA cycle dysfunction, as well as increased inflammatory reaction [48]. The accumulation of succinate in the TCA cycle presents in the heart during ischemia, and its rapid oxidization induces the enhanced mitochondrial ROS production as the result of complex I-involved reverse electron transport (RET) during early reperfusion [6,73,74,75]. These highlight that the redox response during ischemia in stimulated cardiomyocytes is an indispensable element for the process of cell injury during reperfusion [76,77].

Aging is a complicated and progressive process that involves alterations in both physiology and metabolism in every organ and system [77,78]. Compared to young adults, elder patients have diastolic dysfunction with a lower ratio of the early to late ventricular filling velocities (E/A) and a longer left ventricular isovolumetric relaxation time (LVRT) [79]. In addition, the cardiac systolic function which presents by ejection fraction (EF) and fractional shorting (FS) show a significant decrease in response to I/R stress along with aging [80,81]. The hearts lose the capacity to recover from ischemia in clinical settings with a lower survival rate in old patients [82]. Animal experiments also showed blunted functional recovery and enlarged infarct size following I/R in the aged heart [36,83,84]. Thus, it is critical to understand the mechanisms of the increased myocardial damage caused by I/R in the aged heart in order to develop potentially effective strategies for myocardial protection.

Diminished mitochondria electron transport chain (ETC) activity and elevated ROS production contribute significantly to the pathogenesis of aging hearts, leading to increased oxidative damage, including lipid peroxidation and mtDNA damage [22,85,86,87,88,89]. Thus, mitochondria, as the main source of ROS, are a potential cause for the increased injury in the aged heart [90,91,92]. A previous study demonstrated that ischemic damage increased the formation of oxyradicals conducted by complex III in the aging heart which overlapped with the pre-existing aging defects [91]. Increased myocyte apoptosis and the oxidative modification of mitochondrial proteins also supports the greater mitochondria-derived oxidative damage in the aged heart during I/R [93,94]. Moreover, the impairment of mitochondrial OXPHOS and the excessive ROS with aging during myocardial I/R exacerbates impaired metabolic flexibility [93], resulting in more severe contractile dysfunction [95] and the intolerance to I/R stress in the aged heart [36,84,93]. These findings prompt a close relationship between ROS and age-related cardiac dysfunction upon I/R stress, and an effective therapeutic management in substrate metabolism is essential to protect myocardium from I/R injury, in particular for the elderly. However, a recent study has observed elevated levels of substrate metabolites but no transcriptional changes in energy metabolic pathways in early heart failure [96]. It suggests that the post-translational modifications contribute to the alterations in energy metabolism that occur in the early stages of heart disease. As a major post-translational modification for cellular signaling, protein acetylation and deacetylation is regulated by deacetylase, such as sirtuin proteins. The activity of sirtuins is coupled with the cellular NAD^+^ level, indicating their close relationship between cellular energy and redox status. Thereby, we will mainly focus on the deacetylase functions of longevity sensor SIRT1 and SIRT3 via modulating redox homeostasis in response to cardiac I/R stress.

## 4. Role of SIRT1 in the Redox Homeostasis during Myocardial I/R

SIRT1 is the closest mammalian homolog to the yeast Sir2 protein in sequence and is expressed throughout the body, for example, in adipose tissue, liver, heart, and muscle [97]. In the cardiomyocytes, SIRT1 is predominantly located in the nucleus [98]; meanwhile, SIRT1 is also reported to play important roles in cytoplasm and mitochondria fraction [98,99]. Alcendor et al. revealed that the overexpression of SIRT1 in mouse cardiac muscle could protect the heart from oxidative stress and alleviate age-related cardiac hypertrophy [100]. Beyond these, SIRT1 has a pivotal role in repressing inflammation and regulating the metabolic process upon stress stimulation, as well as some putative beneficial effect associated with its activation concerning its role in the lifespan extension [36,98,101]. It is notable that the SIRT1 protein expression level declines with aging in hearts [102]. In addition, its activity is also impaired due to the defected NAD^+^ in aged hearts [103]. More interesting, the reduction of SIRT1 in senescence hearts increases their susceptibility of hearts to I/R injury [20]. These results indicate that SIRT1 is indispensable in aged hearts in response to I/R stress. We next aim to summarize the roles of SIRT1 in regulating ROS-related pathways, which is critical for the modulation of substrate metabolism and inflammatory response in aged hearts during I/R injury.

**Figure 1:**
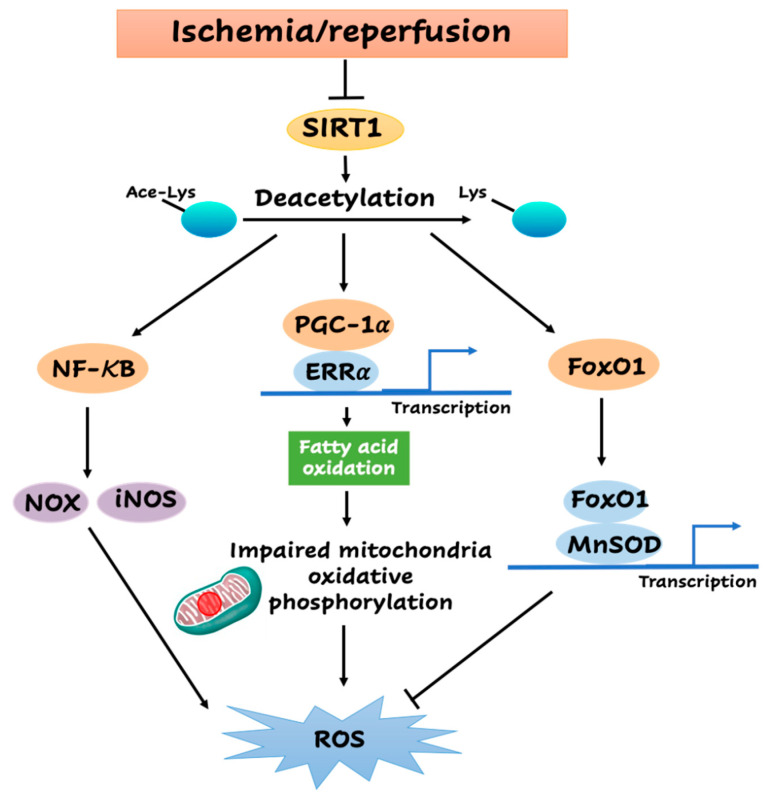
Sirtuin1 (SIRT1)-mediated downstream events associated with reactive oxygen species (ROS) production during myocardial ischemia and reperfusion (I/R).

SIRT1 induces a substrate metabolism shift for ATP production. SIRT1 interacts with and directly deacetylates peroxisome proliferator-activated receptor (PPAR) gamma coactivator-1α (PGC-1α) [104], which is a key switch of mitochondrial biogenesis and fuel usage, to increase its transcriptional activity [105] (Figure 1). As a transcriptional co-activator of the nuclear receptor PPARγ, deacetylated PGC-1α more effectively recruits transcription factor-like estrogen-related receptor α (ERRα), to elevate its synthesis against ROS generation and damage, which are associated with glucose metabolism, fatty acid oxidation, and mitochondrial biogenesis [104,106,107]. PGC-1α also regulates fuel utilization as demonstrated by an ex vivo isolated working heart experiment in which PGC-1α^-/-^ mice exhibited decreased palmitate oxidation with increased glucose oxidation [108]. The findings demonstrated that PGC-1α deficiency may lead to mitochondrial dysfunction and disturbed oxidative metabolism. It is demonstrated that SRT1720, one specific Sirt1 activator, protects the heart from I/R injury through directly increasing the deacetylation of PGC-1α [98]. Collectively, the activation of PGC-1α by SIRT1 and enhanced mitochondria biogenesis may restore energy metabolism in the impaired myocardium and ameliorate I/R injury.

SIRT1 can not only inhibit ROS generation but also influence the antioxidant defense system. FoxO1, an important forhead transcription factor in the cardiovascular system [109], participates in the process of substrate metabolism and cell proliferation [110]. SIRT1 also has the capacity to deacetylase FoxO1 and repress its transcriptional activity during I/R, which acts as a pivotal part in controlling the increase of manganese superoxide dismutase (MnSOD) and inhibiting oxidative stress in cardiac myocytes [111,112] (Figure 1). Consistently, the upregulation of FoxO1 was significantly enhanced with the increased expression of antioxidant MnSOD in cardiac-specific SIRT1 transgenic mice under ischemic stress [111,112]. Moreover, the increase of MnSOD induced by SIRT1 was attenuated in the FoxO1 knockdown tissues [14]. Additionally, the well-recognized immunoresponse-related nuclear factor kappa-light-chain-enhancer of activated B cells (NF-κB) signaling, which is activated during myocardial I/R, can also drive the expression of the antioxidant MnSOD [113].

In addition to mitochondria, the nicotinamide adenine dinucleotide phosphate hydrogen (NADPH) oxidases (NOX) family is another resource for cytoplasmic superoxide radicals generation [114]. As before, NF-κB, a key factor for the immune system, participates in the transactivation of the NADPH oxidase family [115,116]. A substantial amount of literature indicates that the stimulation of SIRT1-related signaling attenuates myocardial I/R injury by inhibiting oxidative damage and inflammatory response [37]. In ischemic cardiomyopathy, these pathophysiological functions of SIRT1 on inflammatory reaction are mainly mediated by the deacetylation of NF-κB [117] (Figure 1). Both animal models and clinical surgery showed that NF-κB is activated by myocardial I/R [118,119], and its inhibition appears to contribute to a reduced infarct size [120]. These findings indicate that SIRT1 may inhibit NF-κB by deacetylation, thereby repressing the ROS generated by the NOX family during I/R. Furthermore, inducible nitric oxide synthase (iNOS) is also upregulated by NF-κB and thus increases the production of ROS [121], suggesting SIRT1 may be involved in the inhibition of cytoplasmic ROS produced by iNOS also. It is notable that aging appeared to be involved in the upregulation of NF-κB and DNA-binding activity in mouse cardiac muscle, which may be even higher in response to I/R stress [122,123]. These findings suggest that the impaired SIRT1 in aged hearts causes the hyperactivation of NF-κB in response to I/R stress, leading to an excessive production of cytoplasmic ROS mediated by NOX and iNOS.

## 5. Role of SIRT3 in the Metabolic Homeostasis during Myocardial I/R

SIRT3 expresses at a high level in the tissues with high metabolic turnover and mitochondrial content, playing a critical effect on the heart and its role in cardiac physiology and pathology [21]. The overexpression of SIRT3 in mouse embryonic fibroblasts reduces cellular ROS by 40% [124]. However, both SIRT3-/- hearts as well as cardiomyocytes cultured from SIRT3-deficient hearts exhibit increased ROS levels [19,125]. These data raise the concept that SIRT3 plays a very important role in the cardiac ROS level. The protein level and activity of SIRT3 also decrease with aging in hearts due to the defected NAD^+^ [20,126,127]. The deficiency of SIRT3 in aging hearts increases their sensitivity to ischemic insults and I/R injury [19]. Thus, understanding the mechanism of SIRT3 in regulating ROS-related pathways during myocardial I/R injury is important for revealing its role in aged hearts.

**Figure 2:**
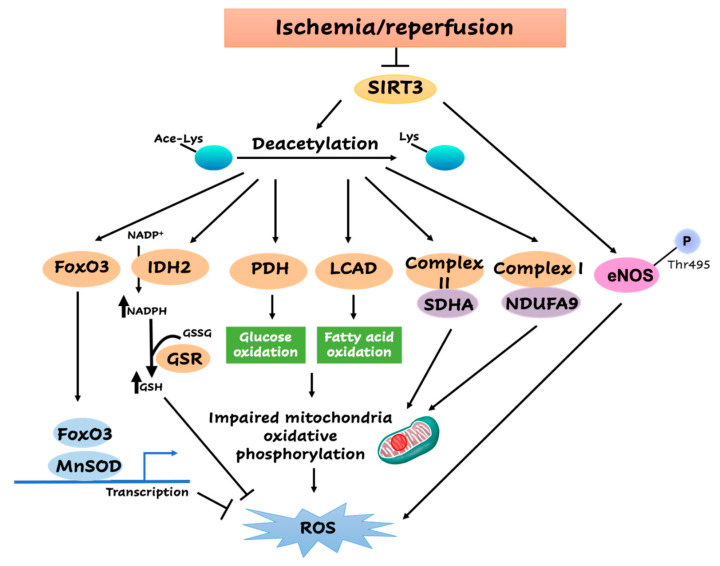
Sirtuin3 (SIRT3)-mediated metabolic downstream events during myocardial ischemia and reperfusion (I/R).

As has been shown, plenty of SIRT3 studies revealed its function coupled with cardiac metabolism [128] (Figure 2). In the heart, increased pyruvate dehydrogenase (PDH) protein acetylation with the defected SIRT3 is associated with the inhibition of its activity [129,130,131]. A few studies have shown that PDH contributes to ROS generation by controlling the glucose metabolism step from pyruvate to acetyl coenzyme A (acetyl-CoA) first entering TCA cycle [132,133]. In isolated rat hearts, the low ATP/Adenosine diphosphate (ADP) ratio during ischemia limited the phosphorylation of PDH and kept only 45% PDH activation of the total enzyme content [134]. The impairment of PDH activity upon early reperfusion may due to the observed high levels of NADH and acetyl CoA and then returned slowly after following reperfusion [134,135,136]. It also has been implicated that the attenuated SIRT3 would lead to the hyperacetylation of long-chain acyl CoA dehydrogenase (LCAD) to the suppressed activity in heart and aging liver [99,126]. LCAD controls the first entry of acetyl CoA generated by fatty acid oxidation to the TCA cycle and assists ROS generation. During the reperfusion period, fatty acid oxidation recovers quickly and dominates as a source of oxygen consumption, as well as ROS generation. Due to the essential roles of PDH and LCAD in modulating glucose and fatty acid oxidation and the following contribution to ROS generation, the role of SIRT3 involved in deacetylating PDH and LCAD could be a way to control the metabolic balance and ROS generation during myocardial I/R. However, Muoio group recently reported that SIRT3 deletion has no impact on mitochondrial respiratory function [137] but may alter the local redox environment.

On the other hand, Lombard et al. implicated that SIRT3 is the leader that controls mitochondrial deacetylation, since the significant hyperacetylation of a wide range of mitochondrial proteins was observed in SIRT3-deficient mice [32]. SIRT3 has the capacity to deacetylase complex I subunit NDUFA9 and complex II subunit SDHA in the mitochondria ETC and augment its activity to maintain the ATP level [138,139] (Figure 2). The deletion of SIRT3 increased the sensitivity to I/R injury and showed increased ROS leakage out and a lowered level of cellular ATP [20,140]. These indicate that SIRT3 could maintain the redox and energy homeostasis through the direct deacetylation of mitochondria ETC complex members. Previous research demonstrated that SIRT3 deacetylates and triggers the enzyme activity of isocitrate dehydrogenase 2 (IDH2), which utilizes NADP^+^ and produces NADPH in the TCA cycle during I/R (Figure 2). The deletion of IDH2 amplifies the liver susceptibility to I/R injury, which is associated with more severe mitochondrial oxidative injury [141,142]. In turn, the upregulation of NADPH is essential for the ROS clearance by mitochondrial glutathione peroxidase (GPX) linked with the process of converting oxidized glutathione (GSSG) into glutathione (GSH). These data suggest that SIRT3 could increase the NADPH level via the deacetylation of IDH2, which increases the GSH and inhibits ROS formation during I/R. In addition, FoxO3, another member of the forkhead transcription factors family, controls cardiac metabolism [143], which is another target for SIRT3-mediated deacetylation [144] (Figure 2). It binds to the gene promoters of MnSOD and induces its transcriptional expression, thereby detoxifying the cellular ROS levels. It is notable that the SIRT3/FoxO3a/MnSOD signaling pathway also plays an important role during I/R in the heart [145,146].

Moreover, macrophages isolated from SIRT3 knockout mice show significant changes in mitochondrial redox homeostasis, which are accompanied by pro-inflammatory-like phenotype alterations as a result of the activation of the NLRP3 inflammasome, as well as the NF-κB pathway [147]. These data implicate that SIRT3 may be involved in the regulation of the cytoplasmic ROS level. One study observed an unchanged phosphorylation at endothelial NOS (eNOS) Ser1177, which is replaced by a decreased phosphorylation of eNOS Thr495 following the transient deletion of SIRT3, which is equivalent to an increased enzymatic activity [148]. SIRT3 deficiency may cause a compensatory effect that is secondary to an increased mitochondrial ROS accumulation. Thus, the increased eNOS activity does not generate an increase in NO upon SIRT3 deficiency. However, the increased eNOS coupling may contribute to counteract increased ROS levels upon SIRT3 deficiency [148]. However, several questions still need to be addressed to fully understand the function of SIRT3 on cytoplasm ROS level upon myocardial I/R.

## 6. Conclusions

Taken together, the balance between the synthesis and clearance of ROS is crucial to maintain healthy homeostasis of the cardiomyocytes under both physiology and I/R stress. Aging markedly increased the damage induced by I/R injury with more severe cardiac dysfunction and myocardial infarction due to the increased free radicals leading to more ROS generation. Age-related excessive ROS production during I/R injury plays a vital role in a series of cellular transductions that lead to mitochondria dysfunction and cardiomyocytes death and finally to severe organ injury.

SIRT1 and SIRT3 have been proposed to be aging-related proteins that mediate the response to I/R stress, especially in aging. Recent studies revealed that defect SIRT1 or SIRT3 increased the sensitivity of hearts to I/R stress as well as enhanced the cardiac ROS level, especially in aged individuals. These indicated that SIRT1 and SIRT3 have robust functions in modulating cardiac ROS production under I/R stress. Notably, ROS and SIRT1/SIRT3 are major regulators of substrate metabolism, which modulates the inflammatory responses during myocardial ischemia and reperfusion. Both metabolic and inflammatory homeostasis are disturbed in aging during the I/R process. It seems that there is an optimal balance between the levels of ROS production and either metabolism or inflammation with the regulation of SIRT1/SIRT3, which confers the most favorable benefits on the protection of the aging heart from more severe I/R injury. Further studies should aim to determine the activity of SIRT1 and SIRT3 on enzyme alterations that drive ROS production in cardiomyocytes involved in the process of IR stress.

Previous findings revealed the discovery of one of the first sirtuin-activating compounds (STACs), resveratrol, via a screen for molecules. Resveratrol increases the activity of human SIRT1 and extends the lifespan of yeast [149]. In addition to SIRT1, resveratrol has been reported to activate SIRT3 and SIRT5, as well as other non-sirtuin targets [10]. After that, several generations of STACs with increasing potency and specificity are generated, including SRT1720 and SRT2104 [150]. STACs bind to the STAC-binding domain in the N terminus of SIRT1 and increase the binding affinity of a substrate for SIRT1 [151]. Honokiol is believed to be a specific SIRT3 activator, although it may also activate SIRT1 [16,152]. It is also necessary to implement the studies with cardiac-specific transgenic mice that inhibit the expression of SIRT1 and SIRT3 in combination with STACs in order to confirm the mechanisms of individual sirtuins. Those studies could contribute to improving the therapeutically effects in a clinical setting and provide appropriate therapeutic approaches for age-related ischemic disease.
